# Matrix metalloproteinase inhibitor, doxycycline and progression of calcific aortic valve disease in hyperlipidemic mice

**DOI:** 10.1038/srep32659

**Published:** 2016-09-13

**Authors:** Jae-Joon Jung, Mahmoud Razavian, Hye-Yeong Kim, Yunpeng Ye, Reza Golestani, Jakub Toczek, Jiasheng Zhang, Mehran M. Sadeghi

**Affiliations:** 1 Section of Cardiovascular Medicine and Cardiovascular Research Center, Yale University School of Medicine, New Haven, CT, United States; 2 VA Connecticut Healthcare System, West Haven, CT, United States

## Abstract

Calcific aortic valve disease (CAVD) is the most common cause of aortic stenosis. Currently, there is no non-invasive medical therapy for CAVD. Matrix metalloproteinases (MMPs) are upregulated in CAVD and play a role in its pathogenesis. Here, we evaluated the effect of doxycycline, a nonselective MMP inhibitor on CAVD progression in the mouse. Apolipoprotein (apo)E^−/−^ mice (n = 20) were fed a Western diet (WD) to induce CAVD. After 3 months, half of the animals was treated with doxycycline, while the others continued WD alone. After 6 months, we evaluated the effect of doxycycline on CAVD progression by echocardiography, MMP-targeted micro single photon emission computed tomography (SPECT)/computed tomography (CT), and tissue analysis. Despite therapeutic blood levels, doxycycline had no significant effect on MMP activation, aortic valve leaflet separation or flow velocity. This lack of effect on *in vivo* images was confirmed on tissue analysis which showed a similar level of aortic valve gelatinase activity, and inflammation between the two groups of animals. In conclusion, doxycycline (100 mg/kg/day) had no effect on CAVD progression in apoE^−/−^ mice with early disease. Studies with more potent and specific inhibitors are needed to establish any potential role of MMP inhibition in CAVD development and progression.

Calcific aortic valve disease (CAVD) is the most common etiology of aortic stenosis^1^. To date, efforts aimed at developing medical therapies to prevent the progression of CAVD have in general failed, and invasive surgical or percutaneous therapies remain the only therapeutic options for aortic stenosis in symptomatic patients. Based on the association of CAVD with dyslipidemia^2^ and aortic valve leaflet lipid accumulation in early CAVD^3^, it is believed that lipids trigger an inflammatory response that promotes valve mineralization. Therefore, lipid lowering with statins appeared as a reasonable strategy for CAVD treatment^4^. Despite promising initial observational studies, statins failed to slow down CAVD progression or improve outcome in randomized clinical trials^5^. Accordingly, the search for effective medical therapies to prevent or slow down CAVD progression in mild to moderate disease continues.

Several matrix metalloproteinases (MMPs) including MMP-1, -2, -3, -7, -9, -12 and -13 are upregulated in CAVD and may mediate tissue remodeling in aortic valve^6^,^7^,^8^,^9^,^10^,^11^,^12^,^13^. Moreover, a recent study showed that MMP activation is significantly increased in early murine CAVD along with valvular inflammation^14^. As such, MMPs could be a therapeutic target for CAVD progression. The antibiotic agent, doxycycline non-selectively inhibits MMP activation and expression, and has been shown to suppress MMP activities in various human cells^15^,^16^,^17^,^18^,^19^,^20^. Preclinical and clinical studies have shown that in the setting of acute ST elevation myocardial infarction, doxycycline may be cardioprotective by reducing adverse left ventricle (LV) remodeling, infarct size and severity, and LV dysfunction^21^,^22^. In murine abdominal aortic aneurysms (AAA) doxycycline attenuates AAA development, especially when the drug is administered before aneurysm induction^23^,^24^,^25^. However, a more recent study found no effect of doxycycline on established murine AAA^26^. Similarly, while doxycycline modulates atherosclerosis in some preclinical and clinical studies, other studies show no effect^25^,^27^,^28^,^29^,^30^. Given the role of MMPs in the pathogenesis of CAVD we investigated the effect of doxycycline on aortic valve function and biology in an established preclinical model of the disease, namely apoE^−/−^ mice on Western diet (WD)^14^. Our data show that despite reaching blood levels found therapeutic in other settings, doxycycline treatment had no significant effect on aortic valve MMP activation evaluated by molecular imaging, function assessed by echocardiography and tissue inflammation.

## Results

**Figure 1:**
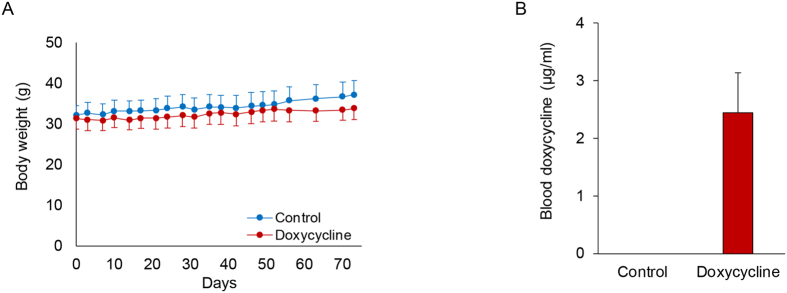
Doxycycline administration in apoE^−/−^ mice. (**A**) Body weight in control and doxycycline-treated apoE^−/−^ mice on high fat diet, measured during the 3 month-period of drug administration. n = 10 in each group. (**B**) Blood doxycycline concentrations measured by high performance liquid chromatography in doxycycline-treated and control animals. n = 2 in each group.

All animals tolerated the administration of doxycycline (100 mg/kg/day) well without body weight loss (Fig. 1A). To confirm that the animals had indeed taken doxycycline, the drug level was measured in a subset of doxycycline-treated and control mice. As expected no drug could be detected in the blood of control animals, while the blood doxycycline level in animals treated with doxycycline was 2.45 ± 0.98 μg/ml (Fig. 1B), a level comparable to previous reports in doxycycline-treated mice and patients taking 200 mg of doxycycline per day^24^,^26^. To determine whether doxycycline administration had any effect on lipid levels, these were measured in a subset of animals in each group. Despite comparable body weights between the two groups, fasting total cholesterol, triglycerides, HDL and LDL were slightly, but not significantly, reduced in the doxycycline group (Table 1).

**Figure 2:**
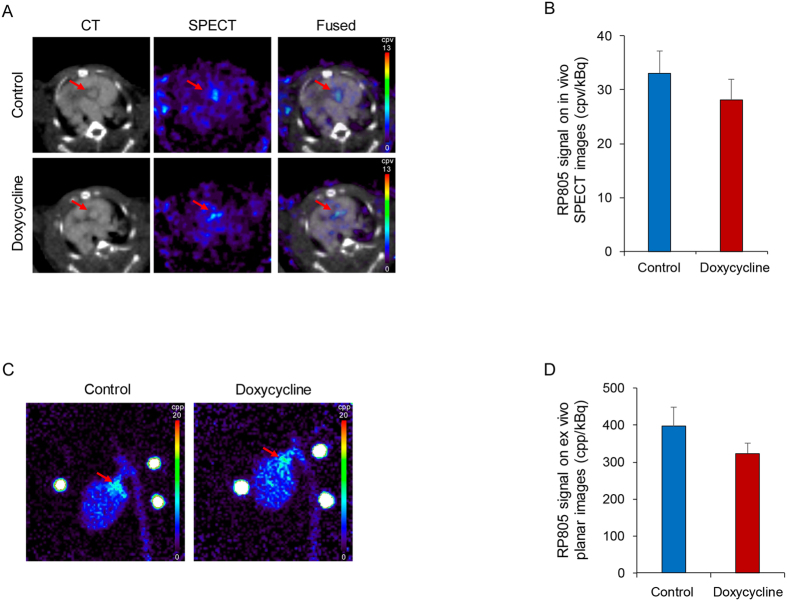
Doxycycline and aortic valve MMP activation in CAVD. (**A**) Examples of *in vivo* CT angiography, RP805 (MMP-targeted) microSPECT, and fused microSPECT and CT transverse images of control (top row) and doxycycline-treated (for 3 months) apoE^−/−^ mice (bottom row) after 6 months of high fat diet. Arrows point to aortic valve area. cpv: counts per voxel. (**B**) Quantification of RP805 uptake in CAVD on *in vivo* SPECT images. n = 8 in each group. (**C**) Examples of *ex vivo* RP805 planar images of the heart and aorta in control (left) and doxycycline-treated apoE^−/−^ mice on high fat diet (right). Arrows point to aortic valve area. cpp: counts per pixel. Three point sources are seen in the field of view. (**D**) Quantification of RP805 uptake in aortic valve area on *ex vivo* planar images. n = 8 in each group.

To investigate the effect of doxycycline on MMP activation, the animals underwent *in vivo* microSPECT/CT imaging using RP805, a ^99m^Tc-labeled tracer with specificity for activated MMPs^14^. Consistent with our previous observations^14^, molecular imaging of MMP activation at 6 months showed uptake of the tracer at the level of aortic valve in control animals (Fig. 2A). A similar signal was detected in animals treated with doxycycline for 3 months (Fig. 2A). Quantification of tracer uptake in aortic valve area showed a 15% (albeit not statistically significant) decrease in MMP signal with doxycycline (33.0 ± 4.1 and 28.1 ± 3.8 counts per voxel/kBq injected dose, respectively for the control and doxycycline groups. n = 8 in each group, Fig. 2B). RP805 uptake in aortic valve was confirmed by *ex vivo* planar imaging (Fig. 2C). Consistent with *in vivo* imaging results, doxycycline administration slightly reduced RP805 uptake in the aortic valve area (19%) without statistical significance (396.5 ± 52.5 and 323.3 ± 29.5 counts per pixel/kBq injected dose, respectively for the control and doxycycline groups. n = 8 in each group, Fig. 2D).

**Figure 3:**
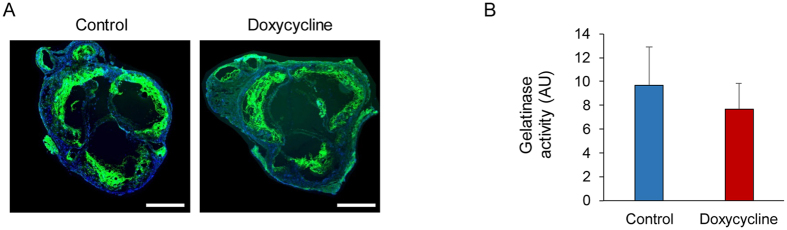
Doxycycline and aortic valve gelatinase activity in CAVD. Examples (**A**) and quantification (**B**) of aortic valve *in situ* gelatinase zymography in control (left) and doxycycline-treated apoE^−/−^ mice on high fat diet (right). MMP activity is in green and nuclei are stained with DAPI in blue. Scale bar: 500 μm. n = 4 in each group.

The effect of doxycycline on aortic valve MMP activity in CAVD was further evaluated by *in situ* gelatinase zymography. Consistent with previous reports^14^, in aortic valve sections from both control and doxycycline-treated mice, gelatinase activity was readily detectable at the valve base and leaflets (Fig. 3A). Consistent with *in vivo* imaging data, gelatinase activity was slightly, but not significantly reduced in animals treated by doxycycline (Fig. 3B). MMP-9 and -12 are amongst the most highly up-regulated genes in CAVD^13^. Therefore, we evaluated the effect of doxycycline treatment on aortic valve MMP-9 and -12 expression by quantitative RT-PCR. Consistent with MMP activation and activity results, the expression of both gene expressions was not affected by doxycycline treatment (Supplemental Figure).

**Figure 4:**
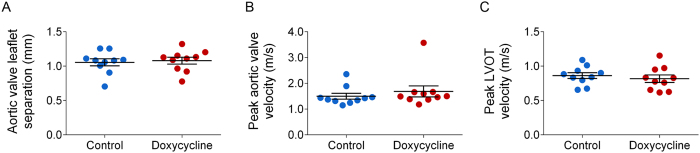
Doxycycline and aortic valve structure in CAVD. (**A**) M mode echocardiography-derived systolic aortic valve leaflet separation. n = 10 in each group. (**B,C**) Echo Doppler-derived peak systolic flow velocity across aortic valve (**B**) and left ventricle outflow tract (LVOT) (**C**). n = 10 in each group.

**Figure 5:**
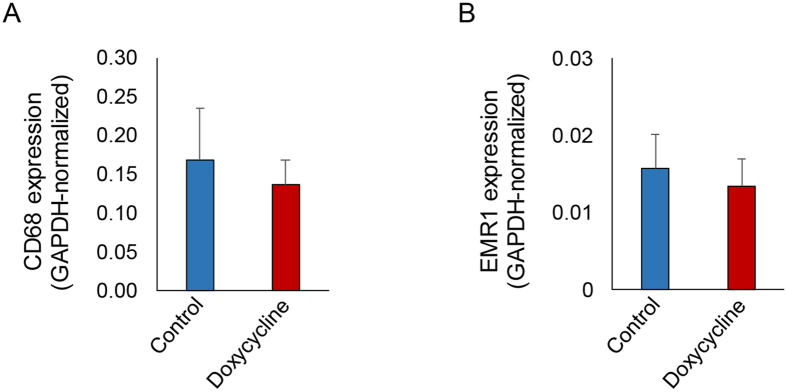
Doxycycline and aortic valve inflammation. Aortic valve GAPDH-normalized CD68 (**A**), and EMR1 (**B**) expression assessed by quantitative RT-PCR. n = 4 in each group.

The effect of doxycycline on aortic valve function *in vivo* was assessed by echocardiography prior to MMP imaging and histological analysis. In the control group, aortic valve leaflet separation and peak flow velocity across aortic valve were similar to those reported in apoE^−/−^ mice fed on WD for 6 months to induce CAVD^14^. In line with MMP imaging data, doxycycline administration had no effect on leaflet separation (control: 1.06 ± 0.05 vs doxycycline: 1.08 ± 0.05 mm. n = 10 in each group, Fig. 4A), peak flow velocity across aortic valve (control: 1499 ± 113 vs doxycycline: 1688 ± 214 mm/s. n = 10 in each group, Fig. 4B) or across left ventricle outflow tract (control: 862 ± 135 vs doxycycline: 817 ± 171 mm/s. n = 10 in each group, Fig. 4C). Next, we assessed the effect of doxycycline on aortic valve inflammation by RT-PCR using two macrophage markers, CD68 and EMR1. Consistent with MMP imaging data, doxycycline did not affect the expression of these macrophage markers (Fig. 5).

## Discussion

Our data demonstrate that doxycycline does not affect CAVD progression in apoE^−/−^ mice with early disease. This lack of effect is reflected in valve biology (MMP expression and activation), histology (inflammation), and physiology (leaflet separation, flow velocity). Given the current absence of effective medical therapies for CAVD, there is great interest in identifying drugs that slow down the progression of CAVD in patients with mild to moderate disease. Potential treatments would target pathogenic pathways and mediators of the disease. However, as shown by the failure of statin therapy, agents deemed promising based on pathophysiology are not necessarily effective in clinical trials^5^. Given the magnitude of the clinical gap it is important to continue to target various aspects of the pathogenic pathways to identify effective drugs. In this regard, evaluation of drugs that are approved for other indications, and which reasonably may have a role in CAVD appears as an important early step.

The hallmark of CAVD is fibrocalcific changes in the aortic valve apparatus, which ultimately lead to restricted opening of the valve. As such, the bone mineralization regulatory pathways, e.g., the wingless-type MMTV integration site family members (Wnt)/β-catenin and bone morphogenetic protein (BMP) signaling pathways are activated in CAVD and appear to play an important role in its development^1^. Inflammation contributes to this process, in part through activation of osteogenic pathways. In addition, inflammatory cells are major sources of MMPs, key mediators of extracellular matrix remodeling required for fibrocalcific alterations of the valve. As such, agents that inhibit MMP expression and activation appear as promising therapeutic agents in CAVD. Several MMP inhibitors have been evaluated in clinical studies. However, due to their significant side effects, the development of these agents has been abandoned and ongoing efforts are focused on developing safer inhibitors, for instance by focusing on specific members of MMP family^31^.

Doxycycline is a broad-spectrum tetracycline antibiotic widely used in the clinic. Besides its antibiotic function, doxycycline has well-recognized anti-MMP properties which are not well defined but appear to be through inhibition of both MMP synthesis (e.g., MMP-2) and activity^18^,^32^,^33^. As such, doxycycline has been used in a number of preclinical and clinical studies to modulate MMP activity: doxycycline therapy reduces MMP-2 and -9 expression and vessel wall inflammation in human abdominal aortic aneurysm (AAA)^32^,^34^,^35^, global and MMP-2/9 activity in left ventricular remodeling after myocardial infarction^21^,^22^, and MMP-1 expression in carotid atherosclerotic plaque^27^. Similarly, in several preclinical models of cardiovascular pathology, doxycycline reduces MMP expression and activity and affects disease progression^36^,^37^,^38^,^39^. However, the effect of doxycycline is not consistent across all studies^26^. The reasons behind this discrepancy are not fully understood but may be related to the animal model, dosing, or stage of the disease^25^,^26^.

There is little data on the effect of doxycycline on cardiovascular calcification. Aortic calcification induced by Vitamin D_3_ or peri-adventitial application of CaCl_2_ was significantly inhibited by doxycycline in Sprague-Dawley rats^40^. Doxycycline also inhibited warfarin and vitamin K-induced aortic calcification in Wistar rats^41^. Unlike these studies we did not observe any effect of doxycycline on valvular inflammation and MMP expression, and a minor, not-statistically significant effect on MMP activation detected by molecular imaging *in vivo* and MMP activity detected by *in situ* zymography *ex vivo*. In line with these findings, aortic valve leaflet separation and peak transvalvular flow velocity were not affected by doxycycline therapy. Given the role of MMPs in the pathogenesis of CAVD, MMP inhibition is expected to inhibit the development and progression of CAVD. While the plasma level of doxycycline obtained in our study (~2.5 μg/ml) is comparable to levels found to be MMP inhibitory in humans and murine studies^24^, tissue levels of the inhibitor in the valve could not be measured. Accordingly, we cannot rule out the possibility that higher doses of doxycycline, a longer course of treatment, alternative MMP inhibitors, or studies in a different species, e.g., humans, may lead to a different result. It is also possible that higher tissue levels are required to modulate valvular calcification and CAVD as compared with arterial calcification. In this regard, it is noteworthy that similar or even lower doses of doxycycline have had an inhibitory effect on arterial calcification in the rat^40^.

To mimic a clinical setting, we opted to test the effect of doxycycline in animals with early stage CAVD. We cannot rule out that this may have affected the outcome of the study. Indeed, in contrast to studies where doxycycline therapy was started before or at the time of AAA induction, in animals with established disease doxycycline has no effect on AAA progression^26^. This would imply that in this model of CAVD the first 3 months of high fat diet triggers a process that cannot be stopped just by targeting MMPs. Alternatively, as we have shown previously, MMP activity in this model peaks after 6 months of high fat diet^14^. While it is easier to show an inhibitory effect by molecular imaging when the MMP signal is maximal, it is possible that MMP inhibition would be more effective in more advanced stages of the disease when MMP activation is less prominent. As such, a later effect of doxycycline, albeit unlikely, cannot be ruled out. Doxycycline is a relatively weak pan-MMP inhibitor, so it is possible that our study with 10 animals in each group is underpowered to detect small changes in aortic valve structure and physiology. Alternatively, given the markedly different temporal expression patterns of specific members of MMP family in CAVD^14^ we cannot rule out the possibility that specific MMP inhibitors would be more effective.

In conclusion, treatment with the pan-MMP inhibitor, doxycycline, had no effect on CAVD progression in mice with early disease, despite reaching therapeutic levels in the plasma. Several factors may have contributed to this lack of effect of doxycycline on murine CAVD, including the necessity of higher doses or longer treatment course. Studies with more potent and specific inhibitors are needed to validate MMPs as targets for preventing CAVD development and progression.

## Materials and Methods

### Reagents

Reagents were purchased from Sigma-Aldrich (St. Louis, MI) unless otherwise specified. RP805, a ^99m^Tc-labeled tracer with specificity for activated MMPs^42^, was synthesized in-house and labeled as described^14^.

### Animal model

Twenty 4 to 5-week-old apoE^−/−^ mice (60% female, originally from Jackson Laboratory, Bar Harbor, ME) were fed a high fat Western diet (WD) (0.15% cholesterol and 40% calories from fat, Harlan Teklad, Madison, WI). After 3 months the animals were randomly divided into 2 groups. The treatment group (n = 10) was given doxycycline in the drinking water at a dose of 100 mg/kg/day along with WD for 3 months, while the control group (n = 10) continued WD alone. The concentration of doxycycline in the water was calculated based on average daily water intake of C57BL/6J strain^43^, and the doxycycline containing water was freshly prepared every other day and kept in light-protected water bottles. All experiments were carried out in accordance with the relevant guideline of, and protocols approved by Yale University and VA Connecticut Institutional Animal Care and Use Committees.

### Blood doxycycline and lipid measurement

After 3 months of doxycycline administration, a subset of mice was fasted overnight for blood collection. Blood doxycycline was extracted using methanol and subjected to high performance liquid chromatography using a system equipped with a photo diode array detector at 350 nm (Waters Corporation, Milford, MA). Samples were separated on a C18 column (Phenomenx, 4.6 × 150 mm, 4 μm, 1 ml/min) using a gradient method (A: 0.1% trifluoroacetic acid in water; B: 0.1% trifluoroacetic acid in acetonitrile; from 20% B to 90% B for 15 min). The data were analyzed with Waters Empower 2.0 software. Serum total cholesterol, triglycerides, HDL and LDL concentrations were determined by Yale Mouse Metabolic Phenotyping Center using a Roche COBAS Mira plus spectrophotometer (GMI, Ramsey, MN).

### Echocardiography

Echocardiography was performed as reported previously^14^. Briefly, echocardiographic images were obtained using a Visualsonics Vevo 2100 system with a 30 MHz probe. The animals were anesthetized with 1–2% isoflurane in 100% oxygen and body temperature was monitored using a rectal probe thermometer. Repeated M mode measurements were performed for maximal aortic valve systolic leaflet separation. Flow velocity across aortic valve and left ventricle outflow track were determined using Power Doppler.

### MicroSPECT/CT and *ex vivo* imaging

MicroSPECT/CT imaging was performed as described with minor modifications^14^,^44^,^45^,^46^. In brief, under anesthesia with 1–2% isoflurane in 100% oxygen, 55.2 ± 2.6 MBq ^99m^Tc-labeled RP805 was injected intravenously through a jugular vein catheter. Two hours after tracer injection, animals were imaged using a high-resolution small animal imaging system (Gamma Medica X-SPECT). SPECT images were acquired using the following optimized parameters: ROR = 2.7 cm; 64 projections, 30 sec per projection; 360° rotation; matrix 82 × 82; and 140-keV photopeak ± 10% window. SPECT images were followed by a non-contrast CT followed by CT angiography using ExiTron nano 12000 (80 μL/min for 1 minute, Miltenyi Biotec, San Diego, CA) to locate the aortic valve. To quantify tracer uptake on microSPECT images a 2 × 2 mm cylindrical region of interest (ROI) was drawn at the level of the aortic valve located by CT. Background activity was defined by a ROI drawn immediately anterior to the valve, and the data were expressed as background-corrected mean activity per kBq tracer injected. After completion of *in vivo* imaging, the heart (with aortic valve) and aorta were harvested for *ex vivo* planar imaging using the following parameters: distance 28 mm, image acquisition time 30 minutes. Tracer uptake was quantified in a 2 × 2 mm square ROI drawn over the aortic valve area.

### Gene expression analysis

mRNA was isolated from frozen tissue sections using Absolutely RNA Nanoprep Kit (Stratagene, La Jolla, CA) and reverse transcribed using QuantiTect Reverse Transcription Kit (Qiagen, Valencia, CA) according to manufacturers’ instructions. Real-time polymerase chain reaction (RT-PCR) was performed on cDNA in triplicates using TaqMan gene expression assays (Applied Biosystems, Foster City, CA), for GAPDH (Mm99999915_g1), MMP-9 (Mm00442991_m1), MMP-12 (Mm00500554_m1), CD68 (Mm00839636_g1), and EMR1 (Mm00802529_m1) and an Applied Biosystems 7500 RT-PCR system.

### *In situ* zymography

*In situ* gelatinase zymography was performed using an Enz-Check Gelatinase Assay Kit (Life Technology, Carlsbad, CA) according to the manufacturer’s instructions with minor modifications. Briefly, freshly frozen 5 μm-thick sections (two sections per valve) were incubated with DQ gelatin solution (0.1 mg/ml in PBS) at 37 °C for 30 minutes, and mounted using Prolong Gold Antifade Mountant containing DAPI (Thermo Fisher Scientific). The slides were photographed with a Spot RT3 camera (Diagnostic Instruments, Sterling Heights, MI) mounted on a Zeiss Axiophot fluorescence microscope (Carl Zeiss Microscopy GmbH, Jena, Germany). MMP activity was quantified by measuring background corrected fluorescence within aortic valve tissues using ImageJ software.

### Statistical analysis

Data were presented as mean ± standard error (SE). Data from doxycycline-treated and control groups were compared using Student’s t-test. The level of significance was set at p < 0.05.

## Additional Information

**How to cite this article**: Jung, J.-J. *et al*. Matrix metalloproteinase inhibitor, doxycycline and progression of calcific aortic valve disease in hyperlipidemic mice. *Sci. Rep.* **6**, 32659; doi: 10.1038/srep32659 (2016).

## Supplementary Material

Supplementary Information

## Figures and Tables

**Table 1:** Effect of doxycycline on plasma lipids in high fat-fed apoE^−/−^ mice.

	Control	Doxycycline
Total cholesterol	1036.9 ± 101.1	858.6 ± 64.7
Triglycerides	67.4 ± 17.5	48.9 ± 14.2
HDL	6.5 ± 1.2	5.1 ± 0.1
LDL	235.3 ± 36.9	171.4 ± 23.5

All values are in mg/dL. n = 3–6 in each group.
