# Supplementary material for: Matrix metalloproteinase inhibitor, doxycycline and progression of calcific aortic valve disease in hyperlipidemic mice
Source: Sci Rep. 2016 Sep 13;6:32659. doi: 10.1038/srep32659 (PMC5020643; doi:10.1038/srep32659)

Matrix metalloproteinase inhibitor, doxycycline and progression of calcific aortic valve disease in hyperlipidemic mice

Jae-Joon Jung, PhD[1,2*], Mahmoud Razavian, PhD[1,2*], Hye-Yeong Kim[1,2], PhD, Yunpeng Ye, PhD[1,2], Reza Golestani, MD, PhD[1,2], Jakub Toczek, PhD[1,2], Jiasheng Zhang, MD[1,2], Mehran M. Sadeghi, MD[1,2]

[1] Section of Cardiovascular Medicine and Cardiovascular Research Center, Yale University School of Medicine, New Haven, CT, United States. [2] VA Connecticut Healthcare System, West Haven, CT, United States.

Supplemental Figure

Doxycycline and aortic valve MMP expression in CAVD. GAPDH-normalized MMP-9 (A) and MMP-12 (B) expression assessed by quantitative RT-PCR in aortic valve tissues of control and doxycycline-treated mice on high fat diet. n = 4 in each group.

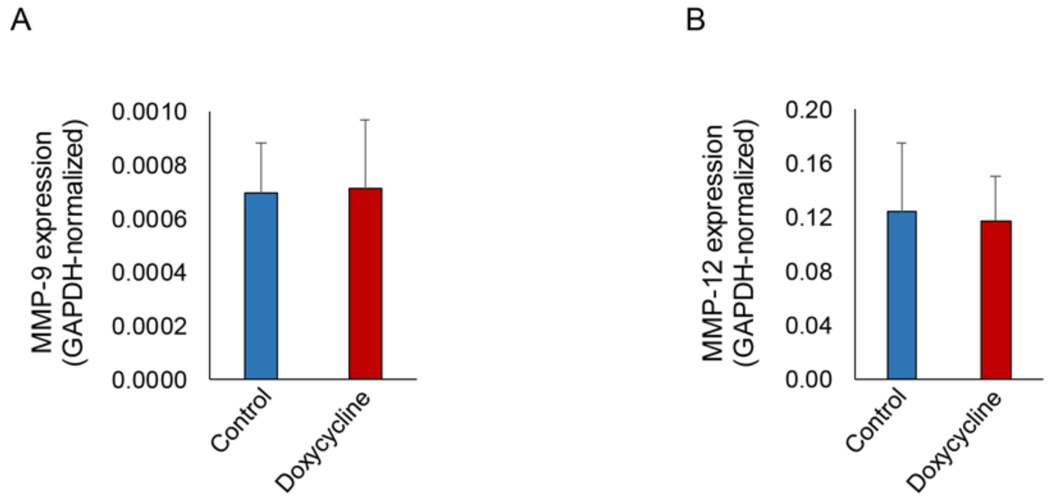

Supplement: Supplementary Information [file srep32659-s1.pdf]
